# Hybrid Superconducting-Ferromagnetic [Bi_2_Sr_2_(Ca,Y)_2_Cu_3_O_10_]_0.99_(La_2/3_Ba_1/3_MnO_3_)_0.01_ Composite Thick Films

**DOI:** 10.3390/ma12060861

**Published:** 2019-03-14

**Authors:** J. Ricardo Mejía-Salazar, José Darío Perea, Roberto Castillo, Jesús Evelio Diosa, Eval Baca

**Affiliations:** 1 National Institute of Telecommunications (Inatel), Santa Rita do Sapucaí-MG 37540-000, Brazil; 2 Institute of Materials for Electronics and Energy Technology (i-MEET), Friedrich-Alexander-Universitaẗ Erlangen-Nürnberg, Martensstrasse 7, 91058 Erlangen, Germany; jd.perea81@gmail.com; 3 Photovoltaic Research Laboratory, Massachusetts Institute of Technology, Cambridge, MA 02139, USA; 4 Facultad de Ciencias Naturales y Matemáticas, Universidad de Ibagué, Ibagué A.A. 730001, Colombia; jesus.castillo@unibague.edu.co; 5 Departamento de Física, Universidad del Valle, Cali A.A. 25360, Colombia; jesus.diosa@correounivalle.edu.co; 6 Centro de Excelencia en Nuevos Materiales (CENM), Universidad del Valle, Cali A.A. 25360, Colombia; 7 Departamento de Física, Grupo de Ingeniería en Nuevos Materiales, Universidad del Valle, Cali A.A. 25360, Colombia

**Keywords:** thick films, composite materials, superconductors, ferromagnetism, manganites

## Abstract

We report here on the development of composite thick films exhibiting hybrid superconducting and ferromagnetic properties, produced through a low-cost, fast, and versatile process. These films were made of high T_*c*_ cuprate superconductor Bi_2_Sr_2_(Ca,Y)_2_Cu_3_O_10_ (with Y:Ca ratio of 5%) and ferromagnetic perovskite La_2/3_Ba_1/3_MnO_3_, synthesized by melting-quenching annealing process on a MgO substrate. Curie temperature for La_2/3_Ba_1/3_MnO_3_ was determined (~336 K ) by magnetic field assisted thermogravimetric analysis (TGA), while superconducting behavior of Bi_2_Sr_2_(Ca,Y)_2_Cu_3_O_10_/MgO films was observed through temperature-dependent resistance measurements. Superconducting features in our hybrid compound were corroborated by temperature-dependent resistivity and magnetic susceptibility.

## 1. Introduction

Precise knowledge of the interplay between mutually antagonistic superconducting and ferromagnetic states is important, not only from the fundamental point of view, in order to understand the interacting mechanism of Cooper pairs (spin-singlet) and ferromagnetism (parallel spin-alignment) [1,2,3,4,5,6,7,8,9], but also for applications in, for instance, magnetic-field-sensors, superconducting-spintronic devices, topological quantum computing, and split-ring resonators made of superconductor for applications in metamaterials [10,11,12,13,14,15]. Such interest has motivated intensive research efforts during the last decades. In particular, composite materials exhibiting superconducting and ferromagnetic features are being actively developed and used as model platforms for these studies, with recent advances including nanodiamonds [8] and superconducting films pierced with ferromagnetic nanorods [9]. However, these latter advances can be seen disadvantageous because of the requirement of expensive and/or complex growing techniques. A simple and cost-effective alternative consists in to exploits similar structural properties of rare earth doped manganites and high T_*c*_ cuprate superconductors to produce hybrid superconducting-ferromagnetic composites, which can be made through a solid state reaction by simple mixing of sample powders [2].

In this work, we use the rare-earth La_2/3_Ba_1/3_MnO_3_ ferromagnetic material, labeled S_1_, and the high T_*c*_ superconductor Bi_2_Sr_2_(Ca,Y)_2_Cu_3_O_10_ (Y/Ca ratio of 5%), labeled S_2_, to produce a composite media exhibiting hybrid superconducting-ferromagnetic features. S_1_ is widely known for the magnetocaloric effect and their colossal magneto-resistance, among other properties, which makes it ideal for high data storage applications [16,17,18,19,20], while S_2_ superconductor has been widely studied in bulk, powder, single crystals, and thin films because of their high critical current [21,22,23], with promising applications for coherent and broadly tunable solid-state quantum THz devices [24,25]. Composite samples were prepared in the form of thick films, synthesized via the fast and versatile melting-quenching annealing (MQA) process [23]. Different sets of measurements, including X-ray difraction(XRD), scanning electron microscopy (SEM) (operated at 30 kV), magnetic field assisted thermogravimetric analysis (TGA), isothermal current-voltage (I-V) curves, resistance and magnetic susceptibility versus temperature, among others, were used to characterize the physical properties of S_1_, S_2_, and (S_2_)_0.99_(S_1_)_0.01_ samples, from where the hybrid features were corroborated.

## 2. Results and Discussion

**Figure 1:**
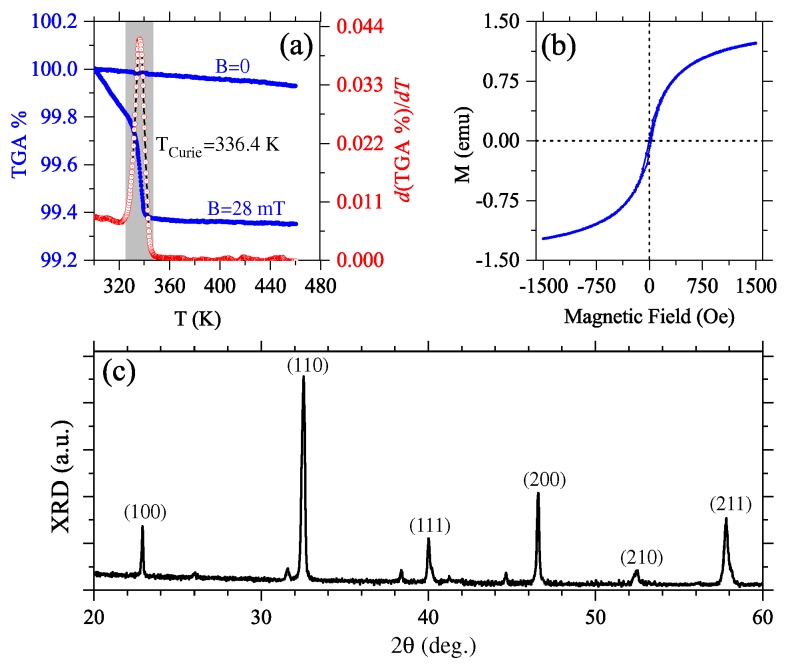
(**a**) Magnetic field assisted TGA measurement of the Curie temperature for S_1_. The peak in the first derivative of the TGA measurement (with B=28 mT) around 336.4 K (shadowed region), corresponds to the Curie temperature. Comparative measurements were made for *B* = 0 and *B* = 28 mT. (**b**) Hysteresis loop for the S_1_ powder sample at 300 K. (**c**) XRD pattern for S_1_.

The rationale in this work is to exploit the similar structural parameters of the rare earth manganite S_1_ and high T_*c*_ S_2_ superconductor to prepare hybrid superconducting-ferromagnetic composite thick films through a low-cost, fast, and versatile process. The physical properties of these hybrid media are determined by their individual mixing components. Ferromagnetic properties of the sample S_1_ are presented in Figure 1. The Curie temperature was measured by TGA, with and without an external applied magnetic field, in the temperature range from 300 to 440 K, as shown in Figure 1a. In contrast with TGA results for *B* = 0, measurements under B=28 mT exhibit three regions: the first one (300–325 K) exhibits a strong linear weight reduction with increasing temperature. The second one, 325–340 K, has an abrupt transition to a stable thermic region, while the third region (>340 K) is characterized by a paramagnetic behavior. The Curie temperature T_Curie_ = 336.4 K, defined at the inflection point of the transition region, was obtained from the peak of the first derivative according to previous reports [17,18]. Results for the hysteresis loop of S_1_ in powder at 300 K, with a coercive field of approximately 2.6 mT (indicating the corresponding ferromagnetic feature), are shown in Figure 1b. XRD pattern for S_1_ is presented in Figure 1c, from where the average grain size of 31.99 nm was obtained through the full width at half-maximum (FWHM) of the diffraction peak by using the Scherrer formula [26].

**Figure 2:**
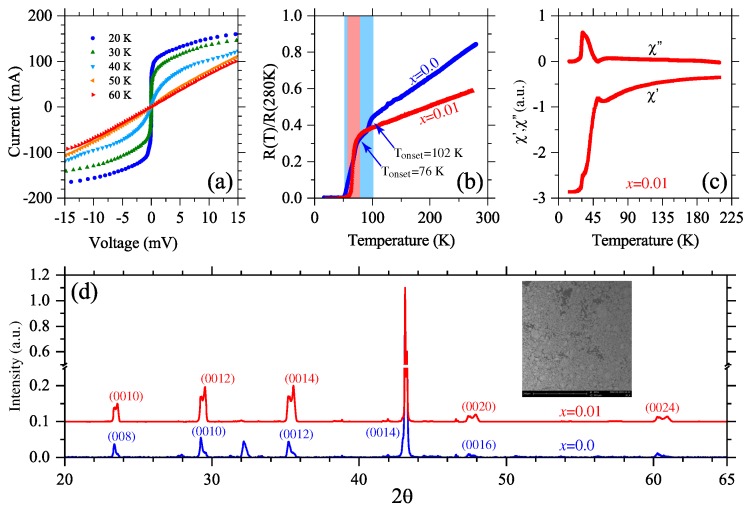
(**a**) Current-voltage curves for S_2_/MgO measured at different temperatures. (**b**) Resistance versus temperature measurements for the samples (S_2_)_1−*x*_(S_1_)_*x*_. Two superconducting critical temperatures can be observed around 76 K and 102 K for x=0.0, in contrast with a single critical temperature around 76 K for x=0.01. The regions of interest are shadowed in figure: 50–102 K for blue region; 50–76 K for red region. (**c**) Real ($\chi^{\prime }$) and imaginary ($\chi^{\prime \prime }$) parts of the magnetic susceptibility as function of the temperature for x=0.01. (**d**) Comparative XRD spectra for x=0.0 and x=0.01. A SEM micrograph of the sample with x=0.01 is presented in the inset of (**d**).

Current-voltage curves for S_2_/MgO measured at 20 K, 30 K, 40 K, 50 K and 60 K are presented in Figure 2a. For temperatures T ≥ 50 K the currents begin to be dominated by normal carriers due to the superconductor-to-conductor transition, which explains the inhibition of supercurrents observed for temperatures below 50 K. This can also be noted from measurements of normalized resistance for S_2_/MgO thick film as function of temperature in Figure 2b, where a metallic behavior is noted for temperatures ranging from T = 100 K to T = 300 K. On the other hand, it is well known from the literature [23] that the BiSrCaCuO system exhibits three superconducting phases (null resistance temperatures), with T_*c*_ = 20 K, 83 K, and 110 K, for the Bi2201, Bi2212, and Bi2223 compounds, respectively. The coexistence of Bi2212 and Bi2223 phases observed from Figure 2b (for x=0) is expected, contrary to their inhibition for x=0.01 (see Figure 2b). The null resistance critical temperatures for the S_2_ and (S_2_)_0.99_(S_1_)_0.01_ samples are T_*c*_ = 51 K and 60 K. The corresponding onset temperatures were measured as 76 K and 102 K, as they are pointed out in the shadowed region of Figure 2b. Such a discrepancy could be due to the fact that S_2_ is (Y-doped)-Bi2223 (at 5%), i.e., some atoms of Ca were replaced by Y. Another possibility, at least in principle, is the influence of diffuse Mn atoms from the perovskite inclusion, which can migrate to Cu sites during the annealing process, significantly depressing their T_*c*_ [27]. The interval between the null resistance temperature (~60 K) and the first transition temperature (ΔT_*c*_ = 42 K), known as the superconductor transition width, allows the coexistence of cooper pairs and normal carriers. In the transition region, resistivity can be described by the Anderson-Kim thermal activated flux-creep model [28], $\rho \left(T,H\right)=\rho_{0}e^{-\frac{U(H)}{kBT}}$, from where the thermal activation energy (for S_2_) of the flux pinning was obtained as U(T,H)≈400 meV. In the case of (S_2_)_0.99_(S_1_)_0.01_/MgO composite thick films, the transition temperature associated to the pure Bi2223 phase (T_*c*_~110 K) is not present. This result is corroborated by temperature dependent magnetic susceptibility measurements in Figure 2c, where the real ($\chi^{\prime }$) and imaginary ($\chi^{\prime \prime }$) parts are presented. The critical temperature for x=0.01 is estimated from these latter results as T_*c*_ = 60 K, in excellent agreement with resistance measurements in Figure 2b. The anomaly observed (for T~35 K), from both curves, could be associated to the paramagnetic Meissner effect (non-zero magnetic moment for temperatures below T_*c*_) due to ferromagnetic particles of S_1_ in the sample. The absence of full Bi2223 phase for (S_2_)_0.99_(S_1_)_0.01_/MgO composite thick films was corroborated from XRD measurements in Figure 2d.

**Figure 3:**
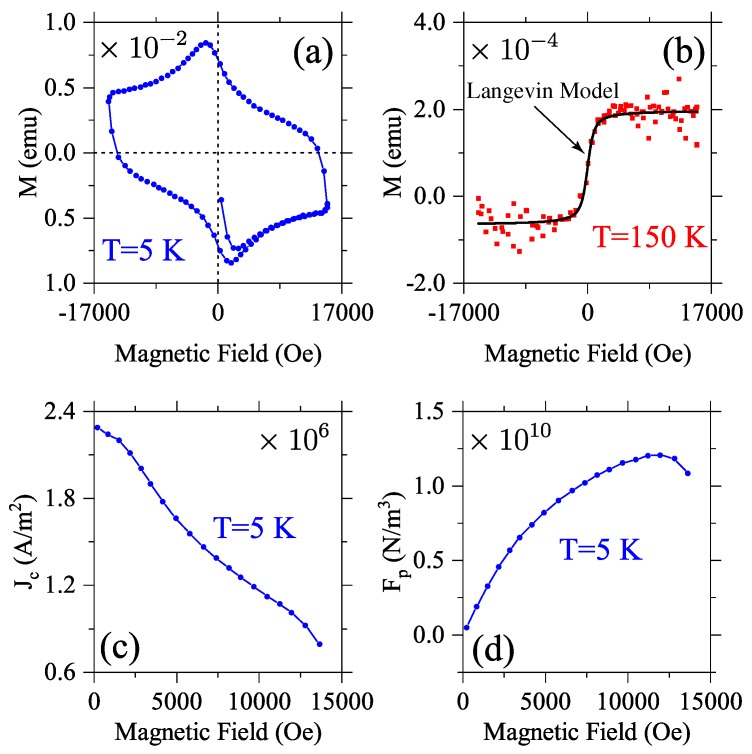
Magnetization versus in-plane magnetic field at (**a**) 5 K and (**b**) 150 K, under zero field cooling regime. Diamagnetic contribution of the MgO substrate was subtracted for results in (**b**). Superconducting current density $J_{c}$ and the macroscopic pinning force density $F_{p}$ are presented in (**c**) and (**d**), respectively.

Magnetic properties of the (S_2_)_0.99_(S_1_)_0.01_ composite are presented in Figure 3. Results for magnetization versus an external in-plane magnetic field, measured for T = 5 K and T = 150 K (under zero field cooling), are presented in Figure 3a,b. A typical superconducting hysteresis loop (diamagnetic behavior with pinned vortices) was observed at T = 5 K, while a ferromagnetic-like behavior is exhibited for T = 150 K. Diamagnetic contribution of the MgO substrate was subtracted from these latter results. Results at 5 K can be described using the model in Ref. [29], where the upper ($M^{+}$) and lower ($M^{-}$) branches of the magnetic hysteresis loop are associated to the superconducting current density through $J_{c}\left(B\right)=\frac{\Delta M}{a(1-a/3b)}$ ($\Delta M=M^{+}-M^{-}$) a=0.5 *μ*m and b=5 mm denote the film thickness and width, respectively. Figure 3c shows the dependence of $J_{c}$ with an applied magnetic field at 5 K. The flux-lines associated with strong pinning can be described by the empirical relation $J_{c}\left(B,T\right)=J_{c}\left(0,T\right)e^{-B/B_{\mathrm{peak}}\left(T\right)}$, where $B_{\mathrm{peak}}$ is the characteristic magnetic field that determines the maximum pinning force. The macroscopic density of pinning force was obtained using the Lorentz formula, $F_{p}=\left|J_{c}\times B\right|$, with numerical results shown in Figure 3d. Solid line in Figure 3b shows a fitting of the experimental results using the Langevin model: $M=M_{0}+M_{s}\left(\coth\frac{H-H_{c}}{H_{s}}-\frac{H_{s}}{H-H_{c}}\right)$, with $M_{0}=6.61\times 10^{-5}$ emu, $H_{c}=-3.3341$ Oe, $M_{s}=-1.3183\times 10^{-4}$ emu, $H_{s}=-338.4265$ Oe.

## 3. Materials and Methods

S_1_ and S_2_ powder samples were prepared separately by solid state reactions explained in detail in Refs. [18,23]. S_1_ and S_2_ powder were mixed according to the composition (S2)_0.99_(S1)_0.01_ and dissolved in acetone to then cover the MgO (001) substrates of 5 mm × 5 mm. A similar process was used to prepare the S_2_/MgO samples. All the samples were melted at 1050 °C for 5 min and then quenched at room temperature in a metallic surface. The resulting films were further annealed at 800 °C for 72 h. Finally, the samples were cooled down to room temperature with a rate of 4 °C/min. We must point out that 1050 °C is the melting temperature of Bi2223 powders on the considered substrate, i.e., the melted time depends of the substrate type, size and thickness. In general, the annealing of Bi leads to absorption of oxygen with an associated rearrangement of the polycrystalline structure. The thicknesses of the films were all estimated around 0.5 micrometers.

The samples were melted and annealed in a tubular furnace MTI Corporation-1200 (Richmond, CA, USA) and characterized by X-ray diffraction in a X’Pert (Royston, UK) with CuK_*α*_-radiation of 1.5409 Å in the range of 20° to 60°. The microstructure morphology of the films were identified using a Jeol/JSM 6490 (Tokyo, Japan). Thermogravimetric analysis was performed in a TA Instruments (New Castle, DE, USA). The electrical properties, R-T and I-V, were measured by the standard four probe technique from room temperature to 12 K via a He closed cycle cryostat Leybold (Cologne, Germany). The electrical measurements were made with a Keithley-2400 Sourcemeter (Cleveland, OH, USA). The ac-magnetic susceptibility measurements were carried out with a Stanford Research model SR830 DSP Lock-In using a homemade ac-susceptometer based in three-coils system. A physical properties measurement system (PPMS) from Quantum Design (San Diego, CA, USA) was used for magnetic measurements.

## 4. Conclusions

(S_2_)_0.99_(S_1_)_0.01_/MgO composite thick films were produced through a cost-effective, fast, and versatile MQA process. Ferromagnetic (S_1_) and superconducting (S_2_) properties of individual components were characterized through different techniques. In the case of composite films, results evidenced a hybrid superconductor-ferromagnetic behavior strongly dependent on the temperature and applied magnetic field, which can be useful in applications for spintronic devices. Classical models were used to obtained the thermal activation energy of the flux pinning, supercurrent densities, and macroscopic density of pinning force.
